# Trapping an Ester Hydrate Intermediate in a π-Stacked Macrocycle with Multiple Hydrogen Bonds

**DOI:** 10.3390/molecules28155705

**Published:** 2023-07-28

**Authors:** Bin Wang, Zi-Ang Nan, Qing Li, Jin Liu, Zi-Xiu Lu, Wei Wang, Zhu Zhuo, Guo-Ling Li, You-Gui Huang

**Affiliations:** 1 CAS Key Laboratory of Design and Assembly of Functional Nanostructures, and Fujian Provincial Key Laboratory of Nanomaterials, Fujian Institute of Research on the Structure of Matter, Chinese Academy of Sciences, Fuzhou 350002, China; wb@fjirsm.ac.cn (B.W.); nanziang@fjirsm.ac.cn (Z.-A.N.); xmliqing@fjirsm.ac.cn (Q.L.); xmliujin@fjirsm.ac.cn (J.L.); zxlu@mail.ustc.edu.cn (Z.-X.L.); wangwei@fjirsm.ac.cn (W.W.); zhuozhu@fjirsm.ac.cn (Z.Z.); 6469@cumt.edu.cn (G.-L.L.); 2 Fujian Science & Technology Innovation Laboratory for Optoelectronic Information of China, Fuzhou 350108, China; 3 Xiamen Key Laboratory of Rare Earth Photoelectric Functional Materials, Xiamen Institute of Rare Earth Materials, Haixi Institutes, Chinese Academy of Sciences, Xiamen 361021, China; 4 University of Chinese Academy of Sciences, Beijing 100049, China

**Keywords:** ester hydrate, self-assembly, π-stacking interaction, hydrogen bonding, intermediate

## Abstract

Ester hydrates, as the intermediates of the esterification between acid and alcohol, are very short-lived and challenging to be trapped. Therefore, the crystal structures of ester hydrates have rarely been characterized. Herein, we present that the mono-deprotonated ester hydrates [CH_3_OSO_2_(OH)_2_]^−^, serving as the template for the self-assembly of a π-stacked boat-shaped macrocycle (CH_3_OSO_2_(OH)_2_)_0.67_(CH_3_OSO_3_)_1.33_@{[ClLCo^II^]_6_}·Cl_4_·13CH_3_OH·9H_2_O (**1**) (L = tris(2-benzimidazolylmethyl) amine), can be trapped in the host by multiple NH···O hydrogen bonds. In the solution of CoCl_2_, L, and H_2_SO_4_ in MeOH, HSO_4_^−^ reacts with MeOH, producing [CH_3_OSO_3_]^−^ via the ester hydrate intermediate of [CH_3_OSO_3_(OH)_2_]^−^. Both the product and the intermediate serve as the template directing the self-assembly of the π-stacked macrocycle, in which the short-lived ester hydrate is firmly trapped and stabilized, as revealed by single-crystal analysis.

## 1. Introduction

Esters are important chemicals because of their wide applications in a variety of products ranging from medicine to biodiesel [1,2,3]. Esters can be produced via esterification reactions between the corresponding acids and alcohols via an intermediate state involving two transition states [4,5]. To trap the short-lived intermediate species, some special strategies have to be adopted. Previous reports have demonstrated that reactive species can be stabilized using the cavities of porous materials [6,7,8,9,10,11,12,13,14,15,16]. For instance, air and moisture-reactive white phosphorus can be safely stored in some tetrahedral cages, as demonstrated by Nitschke et al. and Wu et al. [17,18,19]. Furthermore, various reactive intermediates, including a sulfenic acid [20,21,22], a selenenic acid [23,24], an *S*-nitrosothiol [25,26,27], and a *Se*-nitrososelenol [28], have been isolated for the “peripheral steric protection” of pre-designed molecular cavities [29]. Inspired by the exceptional functionalities of cavities on stabilizing reactive intermediates, Fujita et al. performed a simple and ubiquitous reaction between an amine and an aldehyde in a porous network and successfully observed a trapped transient hemiaminal via single-crystal X-ray diffraction analysis (SCXRD), which usually is a very short-lived intermediate [30].

Different from the above-mentioned strategies, reactive intermediates may also act as the templates for directing the assembly of molecular cages or macrocycles. As a result, the reactive intermediates may be stabilized and trapped by the resulting assemblies, thus allowing structural characterizations. Recently, our group started to using the tripodal ligands tris(2-benzimidazolylmethyl) amine and tris(2-naphthimidazolylmethyl) amine to construct hierarchical assemblies based on π-stacked cages [31,32,33,34,35]. Here, we report the in situ-generated ester hydrate intermediate [CH_3_OSO_2_(OH)_2_]^−^ templates and the assembly of a boat-shaped π-stacked macrocycle, in which the short-lived ester hydrate is firmly trapped and stabilized, thus enabling the structural determination of the intermediate via single-crystal X-ray analysis.

## 2. Results and Discussion

### 2.1. Trapping the Ester Hydrate Intermediate [CH_3_OSO_2_(OH)_2_]^−^

**Figure 1:**
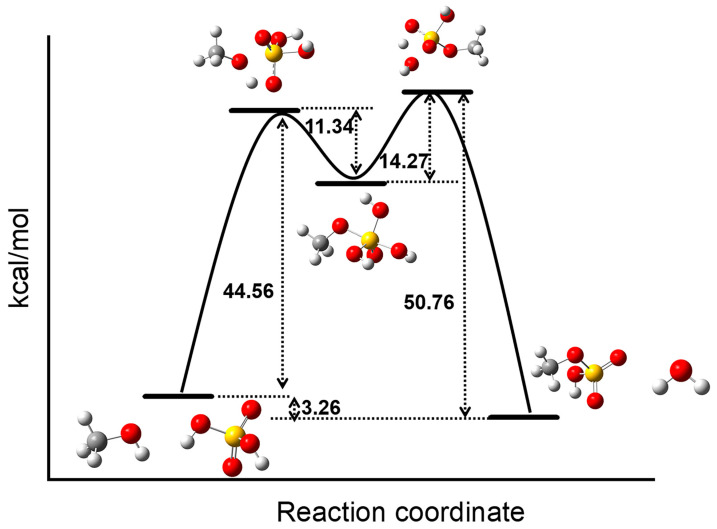
Calculated potential energy surface for the reaction pathway of MeOH and sulfate acid. Although the formation of the ester hydrate intermediate is endothermic, the overall reaction is exothermic.

The esterification between H_2_SO_4_ and MeOH involves the attacking of sulfate acid with a nucleophilic MeOH molecule to form the ester hydrate intermediate CH_3_OSO(OH)_3_ (Figure 1). The ester hydrate intermediate CH_3_OSO(OH)_3_ is short-lived and challenging to be trapped, and, thus, its crystal structure has not been determined. To trap the intermediate [CH_3_OSO_2_(OH)_2_]^−^, we performed esterification between H_2_SO_4_ and MeOH in a MeOH solution containing cobalt chloride hexahydrate (CoCl_2_·6H_2_O), sulfuric acid (H_2_SO_4_), and L. We reasoned that assemblies composed of [ClLCo^II^]^+^ ions can be readily obtained with the templating of the in situ-generated ester hydrate [CH_3_OSO_2_(OH)_2_]^−^. Therefore, the intermediate [CH_3_OSO_2_(OH)_2_]^−^ can be trapped and stabilized due to the confinement of the resulting assembly.

To clarify whether the ester hydrate intermediate [CH_3_OSO_2_(OH)_2_]^−^ was produced and trapped, we performed High-Resolution Mass Spectrometry (HR-MS) analysis on the reactant solution. We successfully identified the species as possibly of {(CH_3_)OSO_2_(OH)_2_@[ClLCo^II^]_3_}^2+^ (identified as {(CH_3_)OSO_2_(OH)_2_@[ClLCo^II^]_3_–H^+^+2Cl^−^–2*e*}^+^) (Figure S1a) and {(CH_3_OSO_2_(OH)_2_)(CH_3_OSO_3_)@[ClLCo^II^]_3_}^+^ (identified as {(CH_3_OSO_2_(OH)_2_)(CH_3_OSO_3_)@[ClLCo^II^]_3_+Cl^−^+H^+^}^+^) (@: guest at host) (Figure S1b). This result suggests the ester hydrate intermediate was possibly produced and trapped.

Keeping the reactant solution undisturbed at 10 °C for seven days, purple cubic crystals of (CH_3_OSO_2_(OH)_2_)_0.67_(CH_3_OSO_3_)_1.33_@{[ClLCo^II^]_6_}·Cl_4_·13CH_3_OH·9H_2_O (**1**) could be harvested. The successful trapping of the ester hydrate [CH_3_OSO_2_(OH)_2_]^−^ was further indicated via single-crystal X-ray analysis of compound **1**. The formula of compound **1** was determined using a combination of single-crystal X-ray crystallography (Table S1) and TG analysis (Figure S2). Single-crystal X-ray analysis of compound **1** revealed a π-stacked boat-shaped macrocycle composed of six [ClLCo^II^]^+^ ions, in which the ester hydrate intermediate [CH_3_OSO_2_(OH)_2_]^−^ and the esterification product [CH_3_OSO_3_]^−^ were trapped in a ratio of 1:2.

**Figure 2:**
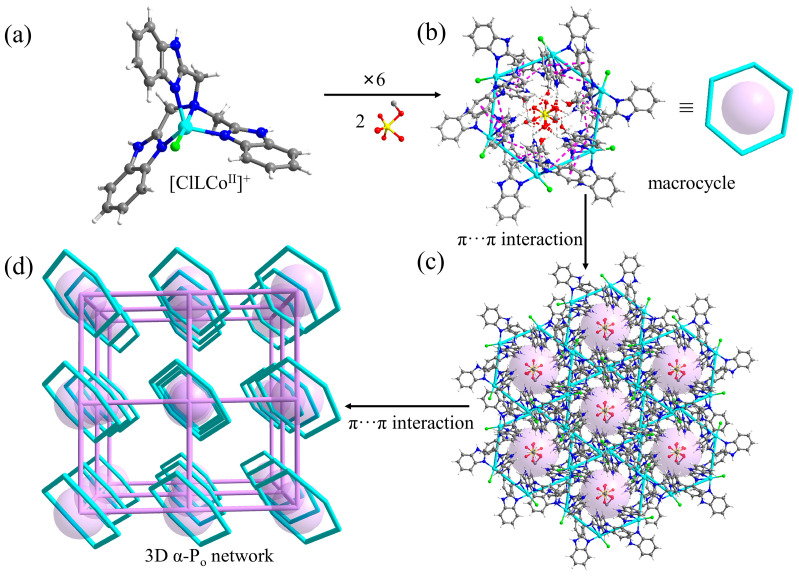
Hierarchical structure of compound **1**. (**a**) Structure of [ClLCo^II^]^+^. (**b**) Top view of the boat-shaped macrocycle of compound **1**. (**c**) View of each macrocycle associating with its six neighbors. (**d**) The 3D α-P_o_ network of compound **1.**

Compound **1** crystallized in the trigonal space group *R*$\overset{-}{3}$, with the asymmetric unit containing one [ClLCo^II^]^+^, 1/9 [CH_3_OSO_2_(OH)_2_]^−^, 2/9 [CH_3_OSO_3_]^−^, 2/3 Cl^−^, 13/6 CH_3_OH, and some disordered H_2_O molecules. In [ClLCo^II^]^+^, the central Co^2+^ bound to four N atoms from the same L ligand and one Cl^−^ in a trigonal-bipyramidal geometry. The tripodal [ClLCo^II^]^+^ featured its three identical benzimidazoylmethyl arms, which were potential active sites for intermolecular π-stacking interactions (Figure 2a). In compound **1**, each [ClLCo^II^]^+^ associated with its two neighbors through *π*···*π* interactions, with centroid distances for phenyl···imidazole, phenyl···phenyl, and imidazole···imidazole of 3.732, 3.809, and 4.044 Å, respectively. As a result, the boat-shaped macrocycle formed. Each vertex of the macrocycle was occupied by [ClLCo^II^]^+^ and each edge was composed of a pair of π-stacked benzimidazoylmethyl arms. Each macrocycle captured two guest molecules via multiple host–guest NH···O (*d*_H···O_ in the range of 1.728–2.141 Å) hydrogen bonds ([(CH_3_OSO_2_(OH)_2_]^−^ and [CH_3_OSO_3_]^−^ existed statistically in a ratio of 1:2) (Figure 2b and Figure S3). Both the [CH_3_OSO_2_(OH)_2_]^−^ and [CH_3_OSO_3_]^−^, located on the *C*_3_-axis, and the [CH_3_OSO_2_(OH)_2_]^−^ intermediate were highly disordered (note: because of the levels of disorder, the interpretation of these data is a hypothesis, with levels of certainty lower than for the remainder of the structure). HR-MS analysis was also performed on the methanol solution of the crystal of compound **1**. The species of {CH_3_OSO_2_(OH)_2_}^−^ (identified as {(CH_3_OSO_2_(OH)_2_+Na^+^+2NH_4_^+^−H^+^}^+^ (Figure S1c) and {(CH_3_OSO_2_(OH)_2_)(CH_3_OSO_3_)}^2−^ (identified as {(CH_3_OSO_2_(OH)_2_)(CH_3_OSO_3_)+3NH_4_^+^+H^+^+*e*}^+^ (Figure S1d) could be identified, which was consistent with the X-ray structure analysis. Therefore, both the [CH_3_OSO_2_(OH)_2_]^−^ intermediate and the [CH_3_OSO_3_]^−^ product were unambiguously produced and trapped. Each macrocycle further associated with its six neighbors (Figure 2c) for the rest of the benzimidazoylmethyl arm, giving rise to a three-dimensional (3D) network. If treating the macrocycle as node and the π-stacked pair of benzimidazoylmethyl arms as linker, the 3D network can be simplified as an α-P_o_ net (Figure 2d). Alternatively, the 3D network can be simplified as a pcu-h net if treating [ClLCo^II^]^+^ as node and the π-stacked pair of benzimidazoylmethyl arms as linker (Figure S4) [36].

### 2.2. Physical-Property Characterization of Compound ***1***

**Figure 3:**
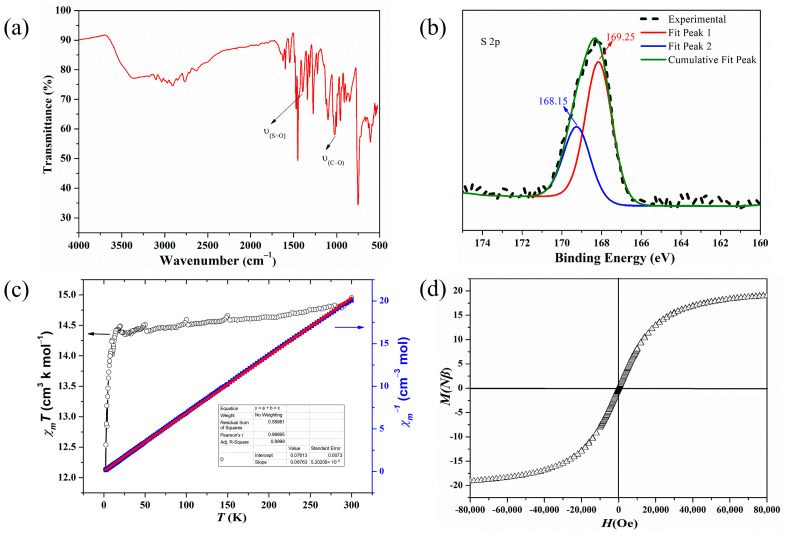
(**a**) IR spectrum of compound **1**. (**b**) XPS of the crystalline sample of **1.** (**c**) The plot of *χ*_m_*T*–*T* and *χ*_m_^−1^–*T* of compound **1**. (**d**) The plot of *M*–*H* of compound **1**.

The phase purity of compound **1** was confirmed via the powder X-ray diffusion pattern (PXRD) measurement (Figure S5). In the infra-red (IR) spectrum of compound **1**, the absorption band at 1395 cm^−1^ can be assigned to υ_(S=O)_ and the band at 1022 cm^−1^ can be assigned to υ_(C–O)_ [37,38] (Figure 3a). The existence of S^VI^ was confirmed using the X-ray photoelectron spectroscopy (XPS) study. The observed peaks at 168.15 and 169.25 eV corresponded to S^VI^ 2p_3/2_ and S^VI^ 2p_1/2_, respectively [39,40] (Figure 3b). A temperature-dependent magnetization study of compound **1** was also performed under 1 kOe field in the 2–300 K range. The *χ*_m_*T* value of 14.95 cm^3^ K mol^−1^ at 300 K was higher than the spin-only value of six isolated high-spin Co^II^ ions (11.25 cm^3^ mol^–1^ K) (Figure 3c) [41,42,43]. This result implies an obvious unquenched orbital contribution. Upon cooling, the *χ*_m_*T* value kept almost constant until 10 K, and then began to decrease, reaching a value of 12.14 cm^3^ mol^–1^ K at 2 K, implying very weak antiferromagnetic couplings between Co^II^ ions. In the range of 300–2 K, the magnetic susceptibility data followed Curie–Weiss law, giving *θ* = –1.13 K and *C* = 14.79 cm^3^ K mol^–1^, confirming the dominant weak antiferromagnetic interactions. To give further insights into the magnetism of compound **1**, the field-dependent magnetizations were measured. The magnetization increased slowly with the increasing field and reached a value of 19.10 Nβ at 80 kOe without obvious hysteresis (Figure 3d), which is consistent with the weak antiferromagnetic couplings between Co^II^ ions [44,45].

## 3. Experimental Methods

### 3.1. Materials and Physical Measurements

The ligand tris(2-benzimidazolylmethyl) amine (L) was synthesized according to the procedure reported in the literature [46], and all the other reagents were commercially obtained and used without further purification. Powder X-ray diffraction (PXRD, Miniflex 600, Akishima, Rigaku, Tokyo, Japan) patterns were performed on a Rigaku Miniflex 600 diffractometer with Cu-Kα radiation using flat plate geometry. High Resolution Mass Spectrometry (HR-MS, Impact II UHR-TOF, Bruker, Billerica, MA, USA) measurements were performed on a DECAX-30000 LCQ Deca XP system. X-ray photoelectron spectroscopy (XPS, Axis Supra, Shimadzu, Manchester, United Kingdom) studies were performed on an AXIS SUPRA Kratos system and the C1s line at 284.8 eV was used as the binding energy reference. Thermogravimetric analysis (TGA/DSC 1, Mettler Telodo, Zurich, Switzerland) was performed on a Mettler-Toledo TGA/DSC 1 system with a heating rate of 10 K/min under an argon atmosphere. Fourier-transform infrared (FTIR, Nicolet iS 50, Thermo Fisher, Waltham, MA, USA) spectra were recorded in the range of 500–4000 cm^−1^ on a Thermo Nicolet is50 FT-IR spectrometer at room temperature. Magnetic measurements (MPMS-5S SQUID, Quantum Designwere, San Diego, CA, USA) were performed on an MPMS-5S SQUID magnetometer.

### 3.2. Synthesis of Compound ***1***

Synthesis of (CH_3_OSO_2_(OH)_2_)_0.66_(CH_3_OSO_3_)_1.33_@{[ClLCo^II^]_6_}·Cl_4_·13CH_3_OH·9H_2_O (**1**): A mixture of L (4.07 g, 0.01 mol), CoCl_2_·6H_2_O (Alfa, 4.76 g, 0.02 mol), and trace amounts of aqueous solution of H_2_SO_4_ (Sinopharm, Beijing, China, 20 μL, 0.001 mmol) in 500 mL methanol (Hushi) was stirred at room temperature for 5 min. After that, the insoluble matters were removed via filtration. The resulting solution was kept undisturbed at 10 °C. Purple cubic crystals of compound **1** were obtained within seven days.

### 3.3. Crystallography

Single-crystal X-ray data were harvested on a Bruker D8 Venture diffractometer with Mo-K_α_ radiation at 200 K. Structures were solved using intrinsic phasing method with SHELXT and refined via the full-matrix least-squares technique on F^2^ with SHELXTL 2014 program [47]. The graphical user interface for the solving and refining process adopted Olex2 software [48]. In the process of solving crystals, some reasonable restriction commands such as DFIX, SIMU, and OMIT were used. All the H atoms were geometrically generated and refined using a riding model. The X-ray crystallographic coordinates for structures reported in this article have been deposited at the Cambridge Crystallographic Data Centre (CCDC) under deposition numbers 2271453. These data can be obtained free of charge from The Cambridge Crystallographic Data Centre via www.ccdc.cam.ac.uk/data_request/cif (accessed on 22 June 2023). Detailed crystallographic data are listed in Table S1.

### 3.4. Computational Methods

We calculated the complete reaction pathway of methanol and vitriol (Figure 1). The geometry structures and the frequency calculations of reactants, products, and intermediate structures, as well as transition-structures (TS) have been optimized using the M062X method and the 6-31G* basis set [49]. Using the same method and basis set, the vibrational frequency calculations were carried out to confirm the local minima intermediate structure and the TS (one negative eigenvalue) on the potential energy surface (PES). The M062X method and the 6-31G* basis set were employed to calculate the intrinsic reaction coordinates (IRCs) [50], verifying that the TS were indeed the lowest saddle points for the expected structure connecting the reaction path. All calculations were performed using Gaussian 16 program [51].

## 4. Conclusions

In conclusion, the reactive ester hydrate intermediate [CH_3_OSO_2_(OH)_2_]^−^ has been successfully trapped and stabilized as the template for directing the assembly of π-stacked boat-shaped macrocycles. The intermediate is firmly trapped in the macrocycle by multiple NH···O hydrogen bonds. This achievement allowed for the structural determination of the intermediate using single-crystal X-ray analysis. This strategy for stabilizing reactive species may be planted to other systems, and, thus, provides a new means of giving insights into the structural transformations that occur during chemical reactions.

## Data Availability

All data related to this study are presented in this publication.

## Supplementary Materials

The following supporting information can be downloaded at: https://www.mdpi.com/article/10.3390/molecules28155705/s1, Table S1: Crystallographic data of the synthetic compound **1**; Figure S1. HR-MS of the reactant solution and the solution of compound **1** in methanol. (a) HR-MS of {(CH_3_OSO_2_(OH)_2_)@[ClLCo^II^]_3_−H^+^+2Cl^−^−2*e*}^+^ identified from the reactant solution. (b) HR-MS of {(CH_3_OSO_2_(OH)_2_)(CH_3_OSO_3_)@[ClLCo^II^]_3_+Cl^−^+H^+^}^+^ identified from the reactant solution. (c) HR-MS of {(CH_3_OSO_2_(OH)_2_)+Na^+^+2NH_4_^+^ −H^+^}^+^ identified from the solution of compound 1 in methanol. (d) HR-MS of {(CH_3_OSO_2_(OH)_2_)(CH_3_OSO_3_)+3NH_4_^+^+H^+^+*e*}^+^ identified from the solution of compound 1 in methanol; Figure S2: TGA for compound 1; Figure S3. Side view of the structure of the boat-shaped macrocycle templating by [CH_3_OSO_2_(OH)_2_]^−^ and [CH_3_OSO_3_]^−^ in compound **1**. (inset: structure of the ester hydrate [CH_3_OSO_2_(OH)_2_]^−^ in compound **1**.); Figure S4. The pcu-h network of compound **1** when treating [ClLCo^II^]^+^ as node and the π-stacked pair of benzimidazoylmethyl arms as linker; Figure S5: PXRD patterns of compound **1**.

## Sample Availability

Samples are available from the authors.
