# Supplementary material for: Trapping an Ester Hydrate Intermediate in a π-Stacked Macrocycle with Multiple Hydrogen Bonds
Source: Molecules. 2023 Jul 28;28(15):5705. doi: 10.3390/molecules28155705 (PMC10420806; doi:10.3390/molecules28155705)

checkCIF/PLATON report

Structure factors have been supplied for datablock(s) w

THIS REPORT IS FOR GUIDANCE ONLY. IF USED AS PART OF A REVIEW PROCEDURE FOR PUBLICATION, IT SHOULD NOT REPLACE THE EXPERTISE OF AN EXPERIENCED CRYSTALLOGRAPHIC REFEREE.

No syntax errors found.   CIF dictionary   Interpreting this report

Datablock: w

```
Bond precision:   C-C = 0.0083 A           Wavelength=0.71073

Cell:              a=22.8534(6)      b=22.8534(6)      c=28.8133(11)
                   alpha=90          beta=90           gamma=120
Temperature:       200 K
```

	Calculated	Reported
Volume	13032.4(8)	13032.4(8)
Space group	R -3	R -3
Hall group	-R 3	-R 3
Moiety formula	18(C24 H21 Cl Co N7), C2 O10 S2, 4(C H3 O4 S), 3(C O), 36(C H4	3(C24 H21 Cl Co N7), 6(C H4 O), C0.667 H2 O2.667 S0.667, 2(Cl),
Sum formula	C477 H534 Cl30 Co18 N126 O65.75 S6	C79.50 H50.92 Cl5 Co3 N21 O10.96 S
Mr	11400.93	1861.76
Dx,g cm-3	1.453	1.423
Z	1	6
Mu (mm-1)	0.814	0.813
F000	5896.0	5668.0
F000'	5909.28	
h,k,lmax	29,29,37	29,29,37
Nref	6660	6657
Tmin,Tmax	0.864,0.907	
Tmin'	0.850	

```
Correction method= Not given

Data completeness= 1.000           Theta(max)= 27.494
```

R(reflections)= 0.0708(4323) wR2(reflections)= 0.2171(6657)

S = 1.027 Npar= 432

The following ALERTS were generated. Each ALERT has the format
test-name_ALERT_alert-type_alert-level.
Click on the hyperlinks for more details of the test.

Alert level B

PLAT043_ALERT_1_B Calculated and Reported Mol. Weight Differ by .. 230.37 Check

Author Response: Due to the disorder of solvent molecules and the compound crystallized in the highly symmetric space group, the hydrogen atoms of some solvent molecules are not identified, so the actual Molecular formula is different from the crystallographic Molecular formula.

PLAT420_ALERT_2_B D-H Bond Without Acceptor O1 --H1BB . Please Check

Author Response: The oxygen atom of methanol (O1) acts as an imidazole group's hydrogen bond acceptor. In the structure, the hydrogen atom of methanol (H1BB) cannot find a suitable hydrogen bond acceptor.

Alert level C

```
PLAT041_ALERT_1_C Calc. and Reported SumFormula    Strings  Differ     Please Check
PLAT068_ALERT_1_C Reported F000 Differs from Calcd (or Missing)...     Please Check
PLAT234_ALERT_4_C Large Hirshfeld Difference O6       --C0AA     .       0.18 Ang.
PLAT243_ALERT_4_C High   'Solvent' Ueq as Compared to Neighbors of         O1 Check
PLAT260_ALERT_2_C Large Average Ueq of Residue Including      O0AA      0.211 Check
PLAT260_ALERT_2_C Large Average Ueq of Residue Including        O1      0.101 Check
PLAT260_ALERT_2_C Large Average Ueq of Residue Including        O2      0.146 Check
PLAT260_ALERT_2_C Large Average Ueq of Residue Including       Cl4      0.123 Check
PLAT260_ALERT_2_C Large Average Ueq of Residue Including      O1AA      0.159 Check
PLAT341_ALERT_3_C Low Bond Precision on  C-C Bonds ...............    0.00833 Ang.
PLAT906_ALERT_3_C Large K Value in the Analysis of Variance ......      2.737 Check
PLAT975_ALERT_2_C Check Calcd Resid. Dens.  0.63Ang From O1AA    .       0.61 eA-3
PLAT975_ALERT_2_C Check Calcd Resid. Dens.  0.50Ang From C1AA    .       0.49 eA-3
PLAT976_ALERT_2_C Check Calcd Resid. Dens.  0.83Ang From O1      .      -0.47 eA-3
PLAT976_ALERT_2_C Check Calcd Resid. Dens.  0.83Ang From O1      .      -0.47 eA-3
PLAT976_ALERT_2_C Check Calcd Resid. Dens.  0.82Ang From O1      .      -0.42 eA-3
```

Alert level G

```
FORMU01_ALERT_1_G  There is a discrepancy between the atom counts in the
            _chemical_formula_sum and _chemical_formula_moiety. This is
            usually due to the moiety formula being in the wrong format.
            Atom count from _chemical_formula_sum:   C79.5 H50.92 Cl5 Co3 N21 O10.
            Atom count from _chemical_formula_moiety:C79 H89 Cl5 Co3 N21 O10.267 S
FORMU01_ALERT_2_G  There is a discrepancy between the atom counts in the
```

```
            _chemical_formula_sum and the formula from the _atom_site* data.
            Atom count from _chemical_formula_sum:C79.5 H50.92 Cl5 Co3 N21 O10.96
            Atom count from the _atom_site data:  C79.5 H89. Cl5. Co3 N21 O10.9582
CELLZ01_ALERT_1_G Difference between formula and atom_site contents detected.
CELLZ01_ALERT_1_G ALERT: Large difference may be due to a
            symmetry error - see SYMMG tests
           From the CIF: _cell_formula_units_Z    6
           From the CIF: _chemical_formula_sum  C79.50 H50.92 Cl5 Co3 N21 O10.96 S
           TEST: Compare cell contents of formula and atom_site data

           atom    Z*formula  cif sites diff
           C        477.00    477.00   -0.00
           H        305.52    534.00 -228.48
           Cl        30.00     30.00   -0.00
           Co        18.00     18.00    0.00
           N        126.00    126.00    0.00
           O         65.76     65.75    0.01
           S          6.00      6.00    0.00
PLAT002_ALERT_2_G Number of Distance or Angle Restraints on AtSite         10 Note
PLAT003_ALERT_2_G Number of Uiso or Uij Restrained non-H Atoms ...         10 Report
PLAT007_ALERT_5_G Number of Unrefined Donor-H Atoms .............           6 Report
PLAT045_ALERT_1_G Calculated and Reported Z Differ by a Factor ...      0.167 Check
PLAT072_ALERT_2_G SHELXL First  Parameter in WGHT  Unusually Large       0.10 Report
PLAT083_ALERT_2_G SHELXL Second Parameter in WGHT  Unusually Large      63.23 Why ?
PLAT172_ALERT_4_G The CIF-Embedded .res File Contains DFIX Records         11 Report
PLAT178_ALERT_4_G The CIF-Embedded .res File Contains SIMU Records          4 Report
PLAT186_ALERT_4_G The CIF-Embedded .res File Contains ISOR Records          3 Report
PLAT188_ALERT_3_G A Non-default SIMU Restraint Value has been used     0.0010 Report
PLAT188_ALERT_3_G A Non-default SIMU Restraint Value has been used     0.0100 Report
PLAT188_ALERT_3_G A Non-default SIMU Restraint Value has been used     0.0100 Report
PLAT188_ALERT_3_G A Non-default SIMU Restraint Value has been used     0.0010 Report
PLAT300_ALERT_4_G Atom Site Occupancy of S3        Constrained at     0.3333 Check
PLAT300_ALERT_4_G Atom Site Occupancy of O3        Constrained at     0.2222 Check
PLAT300_ALERT_4_G Atom Site Occupancy of O3AA      Constrained at     0.1111 Check
PLAT300_ALERT_4_G Atom Site Occupancy of O2AA      Constrained at     0.2222 Check
PLAT300_ALERT_4_G Atom Site Occupancy of C55       Constrained at     0.1111 Check
PLAT300_ALERT_4_G Atom Site Occupancy of S2        Constrained at     0.6667 Check
PLAT300_ALERT_4_G Atom Site Occupancy of O5        Constrained at     0.6667 Check
PLAT300_ALERT_4_G Atom Site Occupancy of O6        Constrained at     0.6667 Check
PLAT300_ALERT_4_G Atom Site Occupancy of C0AA      Constrained at     0.6667 Check
PLAT300_ALERT_4_G Atom Site Occupancy of H0AA      Constrained at     0.6667 Check
PLAT300_ALERT_4_G Atom Site Occupancy of O0AA      Constrained at        0.5 Check
PLAT300_ALERT_4_G Atom Site Occupancy of C1AA      Constrained at     0.1667 Check
PLAT300_ALERT_4_G Atom Site Occupancy of C27A      Constrained at        0.5 Check
PLAT300_ALERT_4_G Atom Site Occupancy of C27B      Constrained at        0.5 Check
PLAT300_ALERT_4_G Atom Site Occupancy of H1BB      Constrained at        0.5 Check
PLAT300_ALERT_4_G Atom Site Occupancy of H1AA      Constrained at        0.5 Check
PLAT300_ALERT_4_G Atom Site Occupancy of H27A      Constrained at        0.5 Check
PLAT300_ALERT_4_G Atom Site Occupancy of H27B      Constrained at        0.5 Check
PLAT300_ALERT_4_G Atom Site Occupancy of H27C      Constrained at        0.5 Check
PLAT300_ALERT_4_G Atom Site Occupancy of H27D      Constrained at        0.5 Check
PLAT300_ALERT_4_G Atom Site Occupancy of H27E      Constrained at        0.5 Check
PLAT300_ALERT_4_G Atom Site Occupancy of H27F      Constrained at        0.5 Check
PLAT300_ALERT_4_G Atom Site Occupancy of Cl4       Constrained at     0.6667 Check
PLAT300_ALERT_4_G Atom Site Occupancy of O1AA      Constrained at       0.25 Check
PLAT302_ALERT_4_G Anion/Solvent/Minor-Residue Disorder (Resd  2  )       100% Note
PLAT302_ALERT_4_G Anion/Solvent/Minor-Residue Disorder (Resd  3  )       100% Note
```

```
PLAT302_ALERT_4_G Anion/Solvent/Minor-Residue Disorder (Resd  4  )       100% Note
PLAT302_ALERT_4_G Anion/Solvent/Minor-Residue Disorder (Resd  5  )        50% Note
PLAT302_ALERT_4_G Anion/Solvent/Minor-Residue Disorder (Resd  7  )       100% Note
PLAT302_ALERT_4_G Anion/Solvent/Minor-Residue Disorder (Resd  8  )       100% Note
PLAT304_ALERT_4_G Non-Integer Number of Atoms in ..... (Resd  2  )       4.67 Check
PLAT304_ALERT_4_G Non-Integer Number of Atoms in ..... (Resd  7  )       0.67 Check
PLAT304_ALERT_4_G Non-Integer Number of Atoms in ..... (Resd  8  )       0.04 Check
PLAT311_ALERT_2_G Isolated Disordered Oxygen Atom (No H's ?) .....       O0AA Check
PLAT311_ALERT_2_G Isolated Disordered Oxygen Atom (No H's ?) .....       O1AA Check
PLAT315_ALERT_2_G Singly Bonded Carbon Detected (H-atoms Missing).        C55 Check
PLAT398_ALERT_2_G Deviating  C-O-C   Angle From 120 for O1       .       55.9 Degree
PLAT415_ALERT_2_G Short Inter D-H..H-X       H5       ..H27A     .       1.69 Ang.
                                    1-x,1-y,1-z  =     10_666 Check
PLAT415_ALERT_2_G Short Inter D-H..H-X       H1AA     ..H9       .       2.13 Ang.
                           1/3-x+y,2/3-x,-1/3+z  =      9_554 Check
PLAT417_ALERT_2_G Short Inter D-H..H-D       H1AA     ..H5       .       2.08 Ang.
                                    1-x,1-y,1-z  =     10_666 Check
PLAT432_ALERT_2_G Short Inter X...Y Contact  O1       ..C0AA     .       2.60 Ang.
                                    1-x,1-y,1-z  =     10_666 Check
PLAT432_ALERT_2_G Short Inter X...Y Contact  O1       ..C55      .       2.75 Ang.
                                    1-x,1-y,1-z  =     10_666 Check
PLAT432_ALERT_2_G Short Inter X...Y Contact  N5       ..C27B     .       2.82 Ang.
                                    1-x,1-y,1-z  =     10_666 Check
PLAT432_ALERT_2_G Short Inter X...Y Contact  N7       ..C55      .       2.91 Ang.
                            2/3+x-y,1/3+x,4/3-z  =     15_556 Check
PLAT432_ALERT_2_G Short Inter X...Y Contact  C9       ..C55      .       2.26 Ang.
                            2/3+x-y,1/3+x,4/3-z  =     15_556 Check
PLAT432_ALERT_2_G Short Inter X...Y Contact  C10      ..C55      .       2.77 Ang.
                            2/3+x-y,1/3+x,4/3-z  =     15_556 Check
PLAT720_ALERT_4_G Number of Unusual/Non-Standard Labels .........           9 Note
PLAT779_ALERT_4_G Suspect or Irrelevant (Bond) Angle(s) in CIF ...      36.20 Deg.
              O3AA -S3   -O3      3_565   1_555   1_555 ......     #  147 Check
PLAT779_ALERT_4_G Suspect or Irrelevant (Bond) Angle(s) in CIF ...      36.20 Deg.
              O3AA -S3   -O3      2_665   1_555   3_565 ......     #  150 Check
PLAT789_ALERT_4_G Atoms with Negative _atom_site_disorder_group  #          1 Check
PLAT794_ALERT_5_G Tentative Bond Valency for Co1       (II)      .       1.89 Info
PLAT822_ALERT_4_G CIF-embedded .res Contains Negative PART Numbers          1 Check
PLAT860_ALERT_3_G Number of Least-Squares Restraints ............          71 Note
PLAT883_ALERT_1_G No Info/Value for _atom_sites_solution_primary .     Please Do !
PLAT910_ALERT_3_G Missing # of FCF Reflection(s) Below Theta(Min).          4 Note
PLAT933_ALERT_2_G Number of HKL-OMIT Records in Embedded .res File          2 Note
PLAT978_ALERT_2_G Number C-C Bonds with Positive Residual Density.          3 Info
```

```
   0 ALERT level A = Most likely a serious problem - resolve or explain
   2 ALERT level B = A potentially serious problem, consider carefully
  16 ALERT level C = Check. Ensure it is not caused by an omission or oversight
  74 ALERT level G = General information/check it is not something unexpected

   8 ALERT type 1 CIF construction/syntax error, inconsistent or missing data
  31 ALERT type 2 Indicator that the structure model may be wrong or deficient
   8 ALERT type 3 Indicator that the structure quality may be low
  43 ALERT type 4 Improvement, methodology, query or suggestion
   2 ALERT type 5 Informative message, check
```

It is advisable to attempt to resolve as many as possible of the alerts in all categories. Often the minor alerts point to easily fixed oversights, errors and omissions in your CIF or refinement strategy, so attention to these fine details can be worthwhile. In order to resolve some of the more serious problems it may be necessary to carry out additional measurements or structure refinements. However, the purpose of your study may justify the reported deviations and the more serious of these should normally be commented upon in the discussion or experimental section of a paper or in the "special_details" fields of the CIF. checkCIF was carefully designed to identify outliers and unusual parameters, but every test has its limitations and alerts that are not important in a particular case may appear. Conversely, the absence of alerts does not guarantee there are no aspects of the results needing attention. It is up to the individual to critically assess their own results and, if necessary, seek expert advice.

Publication of your CIF in IUCr journals

A basic structural check has been run on your CIF. These basic checks will be run on all CIFs submitted for publication in IUCr journals (*Acta Crystallographica*, *Journal of Applied Crystallography*, *Journal of Synchrotron Radiation*); however, if you intend to submit to *Acta Crystallographica Section C* or *E* or *IUCrData*, you should make sure that full publication checks are run on the final version of your CIF prior to submission.

Publication of your CIF in other journals

Please refer to the *Notes for Authors* of the relevant journal for any special instructions relating to CIF submission.

PLATON version of 10/05/2023; check.def file version of 10/05/2023

Datablock w - ellipsoid plot

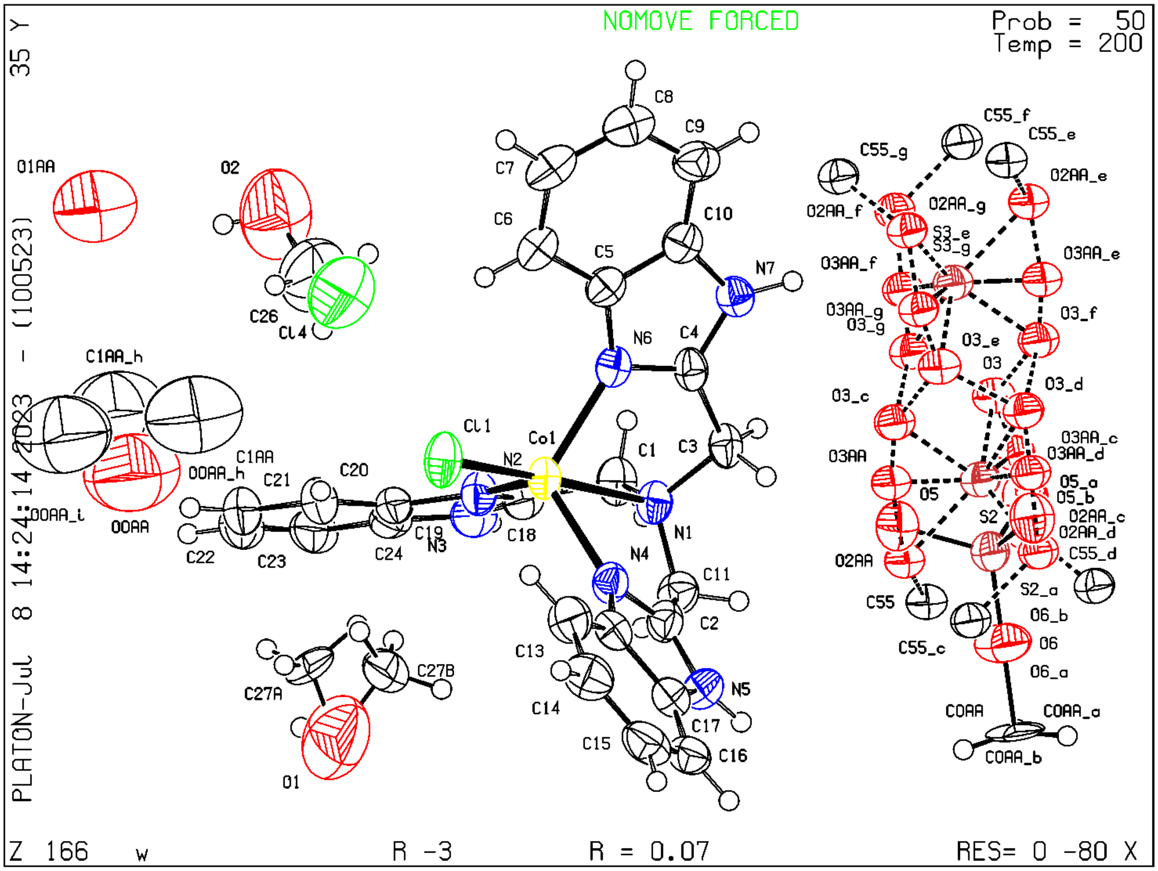

Supplement: Supplementary file 1 [file molecules-28-05705-s001.zip › compound 1 checkcif.pdf]
