# Supplementary material for: Trapping an Ester Hydrate Intermediate in a π-Stacked Macrocycle with Multiple Hydrogen Bonds
Source: Molecules. 2023 Jul 28;28(15):5705. doi: 10.3390/molecules28155705 (PMC10420806; doi:10.3390/molecules28155705)
Supplement: Supplementary file 1 [file molecules-28-05705-s001.zip › Supplementary Information.pdf]

# Trapping an ester hydrate intermediate in a π-stacked macrocycle by multiple hydrogen bonds

Bin Wang[1,2,3,4], Zi-Ang Nan[1,3], Qing Li[1,3], Jin Liu[1,3], Zi-Xiu Lu[1,3], Wei Wang[1,3],

Zhu Zhuo[1,3], Guo-Ling Li[1,3], You-Gui Huang[1,2,3]*

[1] CAS Key Laboratory of Design and Assembly of Functional Nanostructures, and Fujian Provincial Key Laboratory of Nanomaterials, Fujian Institute of Research on the Structure of Matter, Chinese Academy of Sciences, Fuzhou, Fujian, 350002, China.

[2] Fujian Science & Technology Innovation Laboratory for Optoelectronic Information of China, Fuzhou, Fujian, 350108, China.

[3] Xiamen Key Laboratory of Rare Earth Photoelectric Functional Materials, Xiamen Institute of Rare Earth Materials, Haixi Institutes, Chinese Academy of Sciences, Xiamen, Fujian, 361021, China.

[4] University of Chinese Academy of Sciences, Beijing, 100049, China

**Table S1. Crystallographic data of compound 1.**

| Compounds | **1** |
|---|---|
| Formula | $C_{159}H_{203.33}Cl_{10}Co_6N_{42}S_2O_{30.66}$ |
| Formula weight | 3805.60 |
| Temp. (*K*) | 200 |
| Crystal System | Trigonal |
| Space group | $R\bar{3}$ |
| *a* (Å) | 22.8534(6) |
| *b* (Å) | 22.8534(6) |
| *c* (Å) | 28.8133(11) |
| *a* (°) | 90 |
| *β* (°) | 90 |
| *γ* (°) | 120 |
| $V$(Å$^3$) | 13032.4(8) |
| *Z* | 6 |
| $\rho_{cal.}$(g cm$^{-3}$) | 1.423 |
| *μ* | 0.813 |
| *F(000)* | 5668.0 |
| *θ range* (° ) | 2.176–27.494 |
| *Reflections* ($I > 2\sigma$) | 4323 |
| $R_1(I > 2\sigma)$ | 0.0708 |
| $wR_2(all)$ | 0.2171 |
| *GOF* on $F^2$ | 1.027 |
| CCDC# | 2271453 |

[a] $R = \sum||F_0|-|F_c||)/\sum|F_0|$

[b] $wR = |\sum w(F_0-F_c)^2/\sum w(F_0{}^2)^2|^{1/2}$

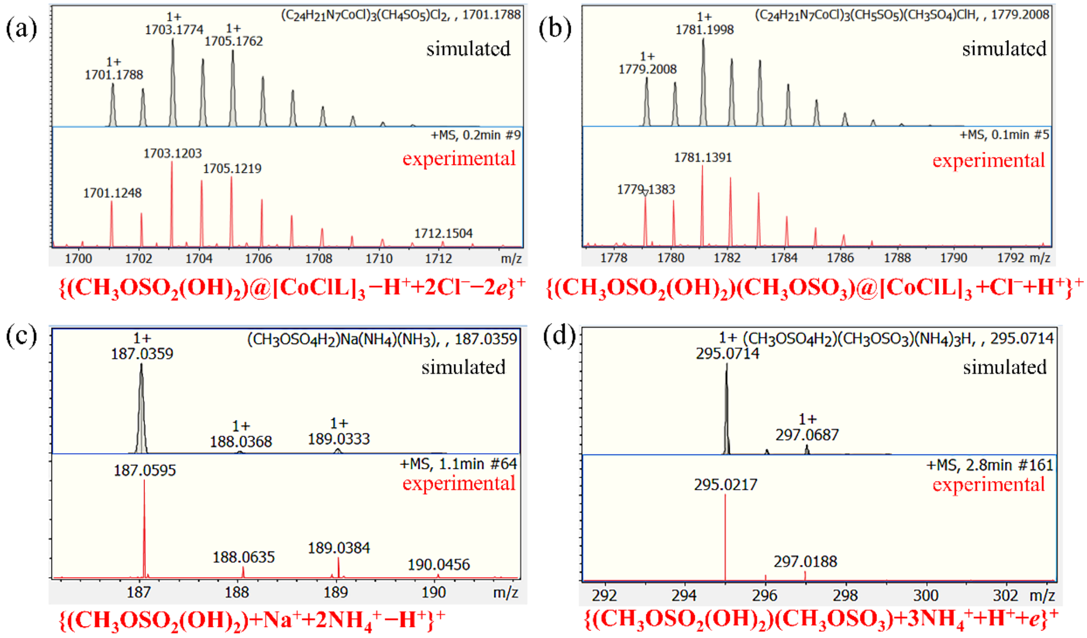


**Figure S1.** HR-MS of the reactant solution and the solution of compound **1** in methanol. (**a**) HR-MS of $\{(CH_3OSO_2(OH)_2)@[ClLCo^{II}]_3{-}H^{+}{+}2Cl^{-}{-}2e\}^{+}$ identified from the reactant solution. (**b**) HR-MS of $\{(CH_3OSO_2(OH)_2)(CH_3OSO_3)@[ClLCo^{II}]_3{+}Cl^{-}{+}H^{+}\}^{+}$ identified from the reactant solution. (**c**) HR-MS of $\{(CH_3OSO_2(OH)_2){+}Na^{+}{+}2NH_4^{+}{-}H^{+}\}^{+}$ identified from the solution of compound **1** in methanol. (**d**) HR-MS of $\{(CH_3OSO_2(OH)_2)(CH_3OSO_3){+}3NH_4^{+}{+}H^{+}{+}e\}^{+}$ identified from the solution of compound **1** in methanol.

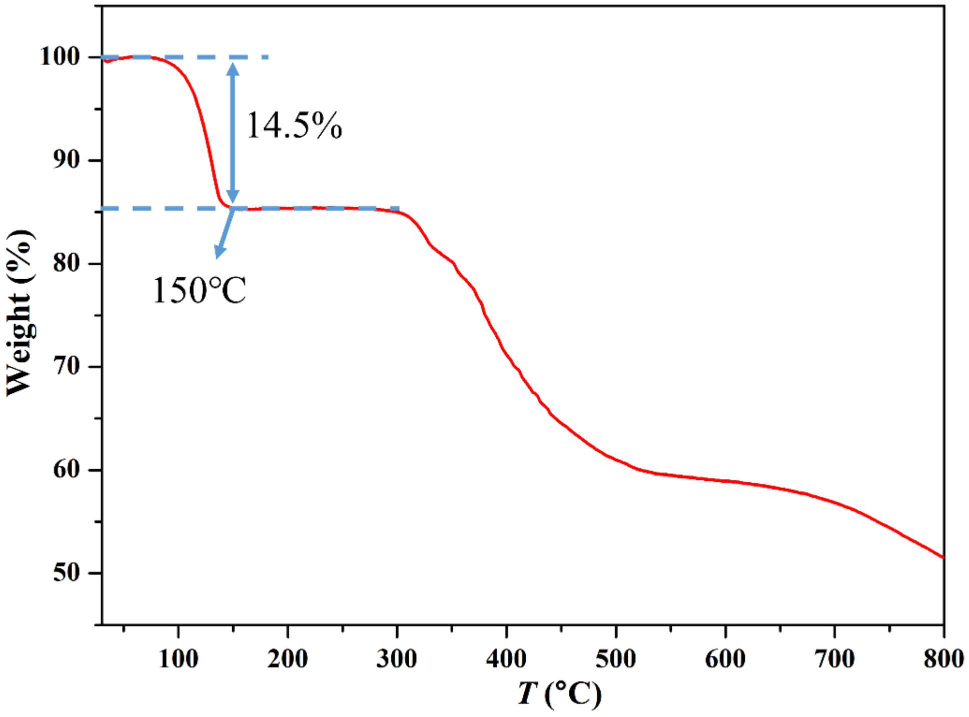


**Figure S2.** TGA for compound **1**.

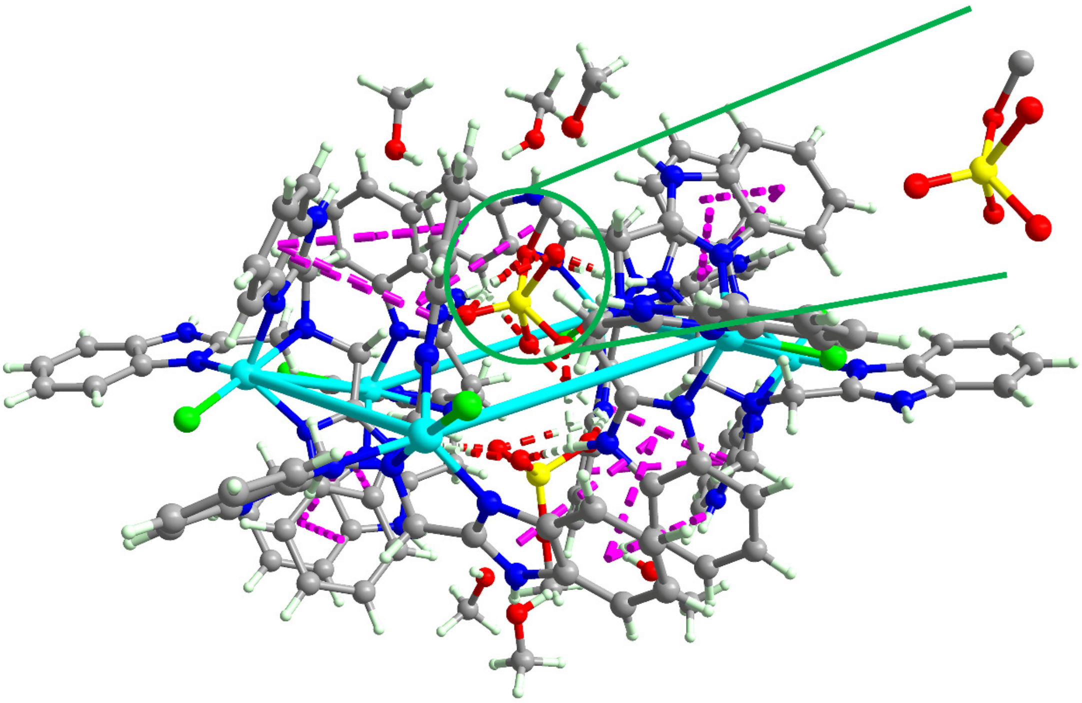

**Figure S3.** Side view of the structure of the boat-shaped macrocycle templating by $[CH_3OSO_2(OH)_2]^-$ and $[CH_3OSO_3]^-$ in compound **1**. (inset: structure of the ester hydrate $[CH_3OSO_2(OH)_2]^-$ in compound **1**.)

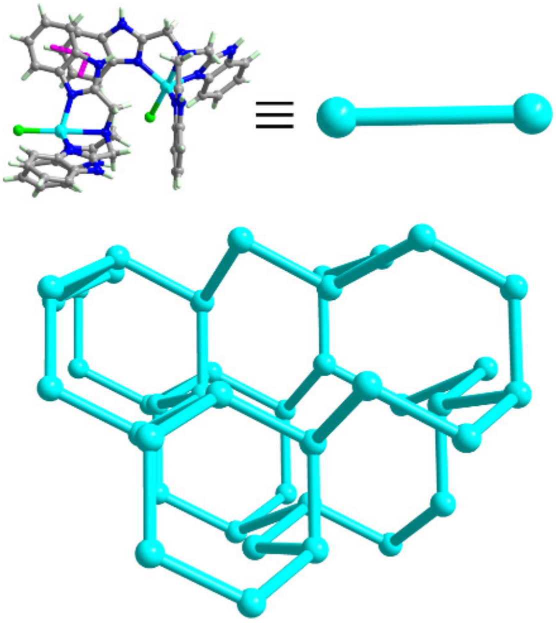

**Figure S4.** The pcu-h network of compound **1** when treating $[ClLCo^{II}]^+$ as node and the π-stacked pair of benzimidazoylmethyl arms as linker.

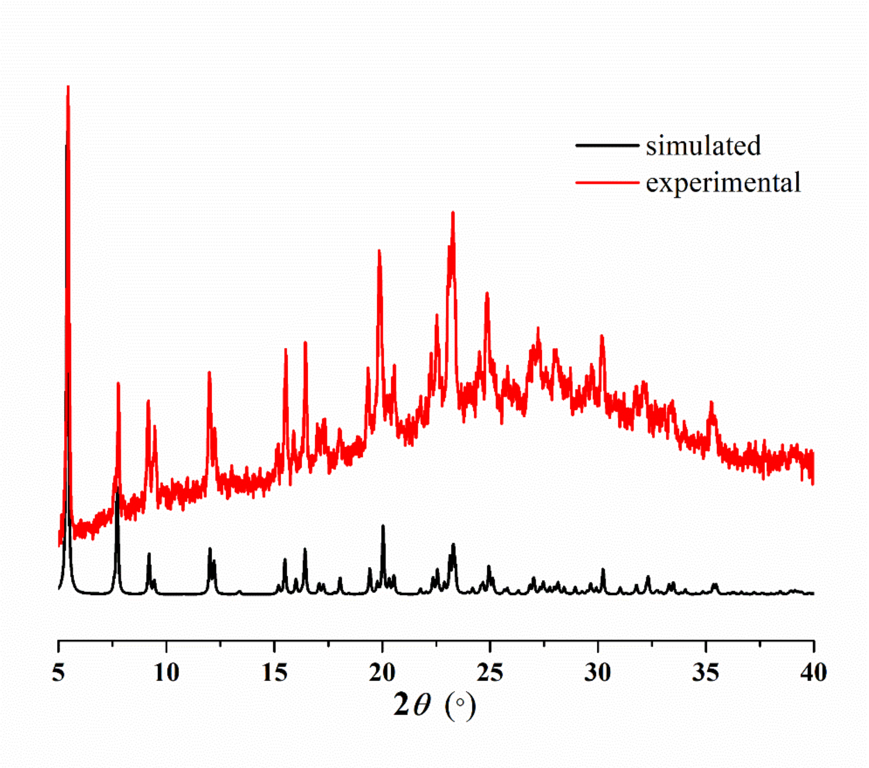


**Figure S5.** PXRD patterns for compound **1**.
